# Dementia and Mild Cognitive Impairment in Prison (DECISION) care pathway and training package: protocol for a realist-informed mixed-methods feasibility study

**DOI:** 10.1136/bmjopen-2025-115466

**Published:** 2026-02-12

**Authors:** Katrina Forsyth, Deborah Buck, Kate Stalker, Victoria Allgar, Jennifer Shaw, Ryan Cowley-Sharp, Rachael Hunter, Charlotte Lennox, Adam O’Neill, Catherine Robinson, Stuart Ware, Louise Robinson

**Affiliations:** 1 Social Care and Society, The University of Manchester, Manchester, UK; 2 University of Plymouth, Plymouth, UK; 3 Peninsula Medical School, Plymouth, UK; 4 Division of Psychology and Mental Health, The University of Manchester, Manchester, UK; 5 Lancashire and South Cumbria NHS Foundation Trust, Preston, UK; 6 Research Department of Primary Care and Population Health, University College London, London, UK; 7 Restore Support Network, Exeter, UK; 8 Secure Services, Lancashire Care NHS Foundation Trust, Preston, UK

**Keywords:** Dementia, Prisons, Feasibility Studies

## Abstract

**Abstract:**

**Introduction:**

Recent research indicates that around 8% of older people living in prison have signs or symptoms of dementia or mild cognitive impairment (MCI), yet the care they receive is not equivalent to care in the community and this means their needs may not be met. We co-developed an intervention specifically for older people living in prison with dementia/MCI (Dementia and Mild Cognitive Impairment in prison care pathway and training package–DECISION). To date, this has not been implemented or evaluated. This paper presents our protocol for a study to assess the feasibility and acceptability of DECISION.

**Methods:**

This is a non-randomised, realist-informed mixed-methods feasibility study with integrated process evaluation, which will take place in two prisons in England. The intervention was codeveloped with experts with lived experience. Participants will include older people living in prison, staff working in prison and peer supporters. We will assess the feasibility and acceptability of the intervention (eg, numbers eligible; rates of recruitment and retention), and the evaluation design (eg, completion rates of standardised outcome measures). Methods will include semistructured, realist-informed interviews; an audit to assess implementation fidelity; focused ethnography; training questionnaires; and collection of resource use data. We will refine the DECISION programme theory using realist-informed methods to examine and refine how contexts and mechanisms interact to produce the intervention’s outcomes.

**Ethics and dissemination:**

This study received a favourable ethical opinion from the Wales REC 3 Research Ethics Committee in January 2025 (reference number 24/WA/0323). HMPPS National Research Committee approval was also granted in January 2025 (reference number 2024-1451). Findings will be disseminated through a range of avenues, including stakeholder engagement events, open-access papers, conference presentations, evidence briefings for commissioners, providers and practitioners, and newsletters for service users.

**Strengths and limitations of this study**

- First protocol for a feasibility study, including process evaluation, examining a new intervention for people living with dementia/mild cognitive impairment in prison.
- Strong patient and public involvement and engagement in the development of the intervention, project proposal and study design.
- Mixed-methods design including interviews, ethnographic observations and an audit of fidelity.
- Taking place in two prisons within the same geographical location.
- Both participating prisons house men only.

## Introduction

 An increase in convictions for historical offences, together with longer prison sentences, has contributed to older people becoming the fastest-growing subgroup among the prison population.^1^,^3^ While older people living in prison tend to age more rapidly compared with their counterparts living in the community^4^ and are more likely to suffer from chronic health conditions,^2 5^ the care they receive is not equivalent to that provided for older people in the community.^6^ This disparity applies not only to physical healthcare but also to support for mental health conditions,^7^ neurocognitive disorders including dementia and mild cognitive impairment (MCI),^8 9^ as well as social care provision.^10^

Previous research suggests that approximately 8% (over one thousand) of older people living in prison in England and Wales may have symptoms of dementia or MCI.^11^ These individuals are often fearful of their environment, reluctant to ask for help and under-diagnosed.^11^,^13^ Prison staff face difficulties in identifying individuals at risk of dementia/MCI and in obtaining appropriate assessments, for example, from Memory Assessment Services.^11^,^15^

Moreover, older people living in prison have substantial social care needs that often go unmet.^16 17^ In particular, dementia/MCI can reduce an individual’s ability to take care of themselves due to memory loss or difficulty communicating.^18^ In England, the Care Act 2014 clarified that local authorities (units of local government) are responsible for the assessment and provision of the social care needs of people living in prison,^19^ and that care should be provided to the equivalent level it is in the community.^20^ There has been limited research regarding the impact of The Care Act in prison,^21 22^ but to date, social care in criminal justice settings has been found to be suboptimal and inconsistent.^16 21 23^

To address the challenges around dementia/MCI and social care needs among older people living in prison, we codeveloped the Dementia and Mild Cognitive Impairment in prison (DECISION) care pathway and training package.^11 24^ The intervention is designed to optimise social care practice in supporting people aged 50 or over with suspected or diagnosed dementia/MCI. Following the development of DECISION, we constructed an initial programme theory (IPT) and logic model (manuscript in press). We subsequently secured funding to implement and evaluate the pathway in the form of a realist-informed feasibility study (DECISION), within two adult prisons in England.

### Aims and objectives

This study aims to explore the feasibility and acceptability of implementing and evaluating the DECISION intervention in two prisons, with a view to developing a plan for a future larger evaluation study. The objectives of the study are to:

1. Assess the feasibility and acceptability of DECISION through realist-informed formative process evaluation.
2. Explore what aspects of DECISION work well, in what contexts, and why, to refine the programme theory.
3. Explore the feasibility and acceptability of the evaluation methods, including use of standardised outcome measures and identification of sources of resource use.

## Methods and analysis

This study protocol is reported in line with the Realist And Meta-narrative Evidence Syntheses: Evolving Standards II (RAMESES II) reporting standards for realist evaluations^25^ together with an adapted version of the Consolidated Standards of Reporting Trials (CONSORT) extension to pilot and feasibility trials.^26^ The adapted CONSORT checklist is provided in online supplemental table S1.

### Study design and setting

**Figure 1:**
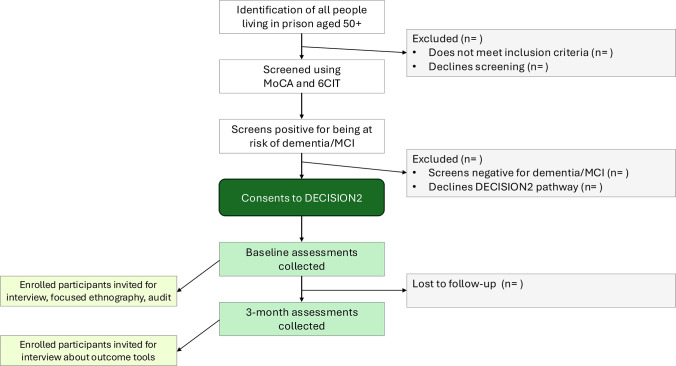
Participant flow. 6CIT, Six-Item Cognitive Impairment Test; MCI, mild cognitive impairment; MoCA, Montreal Cognitive Assessment.

The study is a non-randomised, realist-informed, mixed methods feasibility study with before-and-after standardised outcome measures, integrated formative process and economic evaluations. The study will be conducted over an 18-month period, while individual participants will be followed up for a 3-month period from entry onto intervention. We aim to recruit around 80 participants in total (40 older people onto the care pathway, and approximately 40 staff and peer supporters onto the training element). Recruitment will span an 8 month period. Figure 1 summarises the participant flow for potential recipients of the DECISION intervention, from identification through to 3-month follow-up. The study will take place in two adult male prisons in England.

### The intervention

The DECISION intervention consists of a care pathway and a training package, coordinated and managed by a Dementia Care Coordinator (DCC) who will be a prison-based social worker or other professional with social care training. The DECISION intervention involves screening for people who are at high risk of dementia/MCI; co-ordinating diagnostics relating to dementia/MCI where needed; assessment of social care needs; multi-agency care planning and provision; and dementia awareness and training.

**Figure 2:**
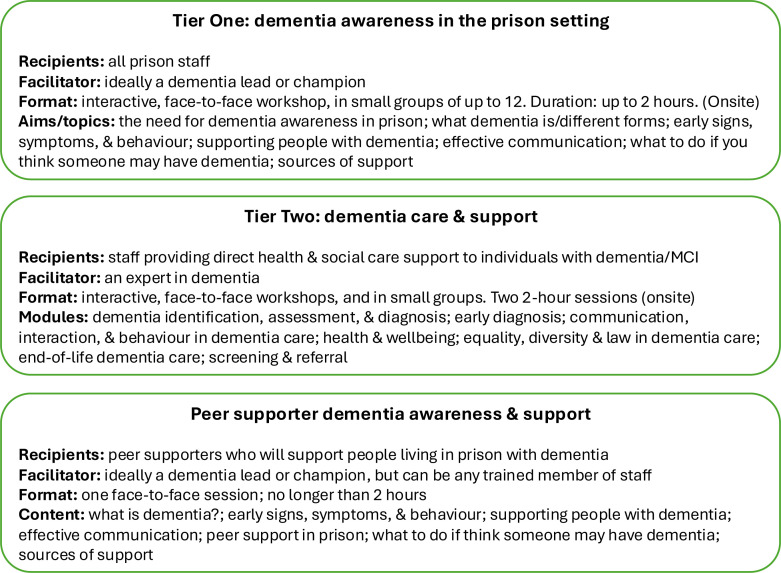
Summary of the content and format of DECISION training packages. MCI, mild cognitive impairment.

The training consists of three packages: Tier 1 is designed for all staff, to enhance their awareness of ageing in prison generally, and the signs and symptoms of dementia/MCI and how to work with them; Tier 2 involves additional, more specialist training for staff who provide direct health and social care support to individuals with dementia or MCI living in prison. The peer supporter training package is designed to improve peer supporters’ awareness of dementia/MCI among people living in prison and to enhance their ability to effectively provide day-to-day support (excluding intimate care). Each package consists of bespoke video case presentations and an accompanying set of slides. Figure 2 summarises the content and format of the training packages.

Staff members and peer supporters will have a minimum of 24 hours to provide consent to participate in the training.

### Participants and sample size

People living with suspected dementia/MCI in prison (PLSDP) will be invited to participate in the DECISION care pathway. Inclusion/exclusion criteria are provided in table 1.

**Table 1:** Inclusion and exclusion criteria for PLSDP

Inclusion criteria	Exclusion criteria
Aged ≥50 years.	Considered by staff not safe to interview alone.
Resident in or newly received into one of the two participating prisons.	Lacking capacity to consent, an appropriate personal or independent consultee could not provide assent.
Scoring below cut-off (22 or lower) on the Montreal Cognitive Assessment and/or Scoring above cut-off (eight or higher) on the Six-Item Cognitive Impairment test.	Does not have a functional command of the English language.

PLSDP, people living with suspected dementia in prison.

Consenting people living in prison, aged 50 and above, will be screened for cognitive impairment by the DCC or research staff, using the Montreal Cognitive Assessment (MoCA)^27^ and the Six-Item Cognitive Impairment Test (6CIT).^28^ A MoCA cut-off score of 22 or lower will be used to identify those with likely cognitive impairment and eligible for the study. This is lower than the general population cut-off, as informed by previous work.^29 30^ A 6CIT score of 8 or higher will be used (a score of 0–7 is considered normal).^28^

Our approach to sampling is based on data obtained from our previous research, which involved screening over 850 people living in prison, using the MoCA.^11^ At the time of writing, 598 older adults are living in the two participating prisons and will be invited to participate in the study. Based on our previous work, we estimate that approximately 5% will refuse to participate, resulting in 568 individuals to be screened for dementia/MCI. Approximately 8% are anticipated to screen positive on the MoCA and/or 6CIT (table 1), resulting in 45 individuals subsequently being offered the DECISION care pathway. Again, based on previous work, we anticipate that 10% will decline participation, hence approximately 40 individuals will receive DECISION. This is in line with guidance which recommends that between 20 and 80 participants be included in feasibility studies.^31^

For the training aspect of DECISION, we estimate that approximately 40 staff (healthcare, social care and prison staff) and ten peer supporters will participate. The peer supporters must already have completed the necessary training as required for their role and be in their position before recruitment. Peer supporters will be excluded if they are not currently in position as a peer supporter/carer/buddy or are due to transfer/be released within the study duration.

### Recruitment and consent

#### Older people living in prison

Prison staff in the two participating prisons will use the Prison National Offender Management Information System to identify all residents aged 50 and over, who meet the inclusion criteria (table 1). The DCC or researchers will then approach and seek their consent to undergo screening for cognitive impairment. Potential participants will be given a minimum of 24 hours to decide whether they wish to take part in the screening. The DCC or researchers will then conduct MoCA and 6CIT tests. Individuals whose screening results on either test suggest cognitive impairment (and therefore possible dementia or MCI (MoCA≤22 or 6-CIT≥8)) will be given at least a further 24 hours to consent to being entered onto the DECISION pathway and to engage in study processes including baseline and follow-up outcome measures.

When we seek consent from individuals to receive DECISION, they will be made aware that they may be approached again to be invited to participate in qualitative interviews and/or ethnographic observation. If approached for interview or observation, they will have a minimum of 24 hours to decide whether to participate in these aspects of the study and provide consent.

Given the low risk of harm posed by the DECISION intervention, withdrawal from the intervention on the grounds of safety or well-being concerns is not foreseen. Participants may choose to withdraw at any stage. In this case, participants will be asked to provide a reason for withdrawal but will be made aware that they are not obliged to give a reason and that their decision to withdraw will not affect their routine treatment. Participants who wish to withdraw from the research will be given the option to either withdraw from participation with the researchers still able to retain their data or to withdraw from participation with their data being destroyed. Participants can request that their data be destroyed up to the point it becomes fully anonymous (ie, we can no longer identify which data is theirs).

Where participants are considered to lack the capacity to consent to participation, researchers will try to identify a ‘Personal Consultee’ as defined by the Mental Capacity Act (MCA).^32^ Researchers will ask if they may contact someone else to advise on the individual’s behalf and will ask the potential participant’s permission to contact the nominated individual. Potential consultees from outside of the prison will only be contacted if the research team can establish that they are aware that the potential participant is in prison and that they have difficulties which limit their capacity to consent. The initial approach to anyone outside of the prison will be made by prison healthcare staff. Personal Consultees will be provided with study information and their role and the reason for them being approached will be explained by researchers. If the potential participant is unable to nominate anyone or give consent to contact someone outside of the prison, researchers will identify an appropriate independent consultee (again, in line with the MCA). This will most likely be a clinician or healthcare worker from within the prison. No pressure will be placed on any individual to act as a consultee and researchers will fully brief consultees regarding the study, to enable them to offer advice on the potential participant’s behalf. If a consultee or nominee advises that the individual would not want to take part, they will not be recruited under any circumstances.

#### Staff and peer supporters

Approximately 40 prison staff members from the two participating prisons will be recruited to receive Tier 1 DECISION training. The head of healthcare, a local authority manager and/or a prison governor or their deputy will identify staff to be invited to receive training. This will include people in prisoner-facing roles such as nurses, social care workers and officers.

Ten peer supporters will be invited to participate in old age and dementia awareness training. Peer supporters to receive DECISION training will be identified by a prison governor or senior officer in consultation with safer custody staff.

Staff and peer supporters will be given a minimum of 24 hours after approach and information to provide consent if they wish to participate in the training sessions and subsequent training questionnaires.

### Feasibility and participant outcomes

To assess feasibility of the intervention and of the evaluation design, we will collect data on the number of older people identified, consented, recruited, screened and eligible for DECISION; retention rates; the number who consent; the number who lack capacity; and reasons for non-retention, where applicable. We will also collect data on completion rates for all measures. A summary of the specific measures and tools to be used, and when, is provided in table 2.

**Table 2:** Outcome measures

	Baseline	3 months
Demographic data	X	
Feasibility data		
Numbers eligible, recruited and screened positively using MoCA/6CIT	X	
Retention and attrition rates		X
Completion rates for all outcome measures		X
Participant outcome/experience measures		
Camberwell Assessment of (met) Need–Research Forensic Version	X	X
Dementia-Related Quality of Life	X	X
The Adult Social Care Outcomes Toolkit	X	X
EQ-5D-5L	X	X
Bespoke DECISION outcome measure	X	X
Bespoke DECISION Fidelity measures		X

6CIT, Six-Item Cognitive Impairment Test; EQ-5D-5L, EuroQol 5-Dimension, 5-Level version; MoCA, Montreal Cognitive Assessment.

PLSDP who agree to participate in the DECISION care pathway will be asked to complete the following questionnaires: Dementia-Related Quality of Life (DEMQoL), which is designed to measure health-related quality of life for people with dementia^33^; the Adult Social Care Outcomes Toolkit (ASCOT), which measures social care quality of life as operationalised by experts by experience^34^; The Camberwell Assessment of (met) Need – Research Forensic Version, which assesses the unmet needs of individuals with mental health problems in prison^35^, and the EQ-5D-5L, which is a generic instrument used to examine health-related quality of life.^36^

However, most of these measures are not designed for people living in prison and our previous experience with outcome measures in this group is that they are often not focused enough on individuals’ personalised needs.^37^ Therefore, we will also co-develop a bespoke DECISION needs assessment outcome measure, with experts by experience, to capture the unique experiences of this group.

If an individual is unable to answer the study questions themselves, proxy versions of the measures will be used where available. Informed consent, or if appropriate, assent from a nominated Personal Consultee, will be obtained before using proxy measures. Where possible, participants will be invited to choose who they would like to act as their proxy.

### Process evaluation

We will conduct a formative, mixed methods process evaluation which will enable us to fully assess the feasibility and acceptability of the intervention to PLSDP and the staff and peer supporters who support them, and to more comprehensively assess the feasibility and acceptability of the study’s evaluation design. We will also evaluate the feasibility of collecting resource use information.

Feasibility and acceptability of the intervention will be assessed through realist-informed semistructured interviews (up to 20 with PLSDP and up to 20 with staff/peer supporters, depending on data saturation), focused ethnography, audit of care plans and notes of all who receive DECISION (subject to consent), and training questionnaires, alongside the data on numbers screened, recruited, assessed and retained; and time taken to deliver the intervention.

Feasibility and acceptability of the evaluation design will be assessed through semistructured interviews and an examination of the contribution of the intervention to intended or unintended outcomes, alongside the completion rates for outcome measures.

### Resource use evaluation

We will evaluate the feasibility of collecting resource use information (eg, details of any care packages; medical, health and social care appointments) from medical records, and through patient-completed resource use and care input questionnaires.

We will use the information from data collected during the study, including audit data on number who attend screening appointments and are MoCA/6CIT positive; recruitment and retention rates; and the time taken to screen, consent and start the intervention. This will be combined with data from refinements to the programme theory and the intervention (see next section), and the information gleaned about ease of delivery of the outcome measures.

### Refinement of the DECISION initial programme theory

We will adopt realist-informed methods to refine DECISION’s IPT and logic model. Realist methodology focuses on ‘what works, for whom, under what circumstances, and how’.^25^ It allows a deconstruction of the causal web of underlying conditions that create effective interventions.^38^,^40^ Realist enquiry is concerned with identifying the underlying generative mechanisms about how interventions do/do not work. A mechanism is both the resource that an intervention provides (eg, training) and the recipients’ response to it (eg, confidence). Realist theories define the underlying causal mechanisms through which outcomes occur, and the contexts in which those mechanisms are activated, expressed as context (C)+mechanism (M)=outcome (O). The CMO configuration is used as the main structure for realist analysis.^40^,^42^ DECISION is a complex intervention,^11^ and the key uncertainties about it include the extent to which it can be implemented as intended in different settings, how it works, and how it should be evaluated.

Refinement will be informed by collating and analysing data from the interviews, focused ethnography, training questionnaires and audit. We will also consider findings from our related scoping review which is exploring issues around screening, assessment and care provision for dementia/MCI among minoritised groups. We will then hold a realist-informed focus group and a final ratification workshop, as outlined below.

#### Realist-informed focus group

Professionals involved in delivering care to participants in receipt of DECISION at both sites (n=12, including GPs, prison psychiatrists, social workers, nurses, Memory Assessment teams, prison staff, etc) and experts with experience of living with dementia/MCI and/or of living in prison will be invited to participate in a focus group to discuss relevant CMOs. These individuals will have at least 24 hours to decide whether they would like to participate and provide consent (or Personal Consultee consent) to be involved in the focus group. We will prompt attendees to discuss the barriers and facilitators when implementing DECISION. This will be audio-recorded and transcribed. The qualitative data analysis process is described in the ‘Analysis Plans’ section and the findings will be used to further refine the logic model and intervention delivery platform.

#### Final ratification workshop

The IPT/logic model and intervention delivery platform will be revised by the research team. Key stakeholders, including academics, practitioners and experts by experience, will be invited to an interactive workshop to review the revised logic model and intervention delivery platform. These individuals will have 24 hours to decide whether they would like to participate and provide consent to be involved in the workshop. This will be the final opportunity to identify potential implementation barriers and to co-create solutions to overcome these. Final changes will then be made to the logic model and intervention delivery platform, ready for implementation in a future study.

### Analysis plans

For quantitative data, statistical analyses will be undertaken using either SPSS or StataSE (V.16 or later), supplemented where required by R. Descriptive statistics will be presented for: participants’ demographic and baseline characteristics; data from the audit and the training questionnaires; numbers eligible, recruited, tested, screened positive and included in DECISION; numbers followed up; retention and attrition rates; and completion rates for all measures. Missing data will be described but not imputed.

Summary statistics of all the collected quantitative outcome and experience measures at each time point will be presented using mean (SD), median (SD) or n (%). Descriptive comparisons between the two time points, including summary statistics of changes in measures between time points, will be presented for all measures. Graphs tracking the trajectory of participants will be used—for example, who became worse, improved or stayed the same. Descriptive statistics of the quantitative outcomes at each time point, and the changes between time points, together with the broad range of data we are also collecting, will allow decision-makers and researchers to explore the potential impact of the intervention.

Qualitative data will be analysed using thematic analysis,^43^ with interpretation informed by realist evaluation principles.^38 42^ Themes will be examined to generate explanatory accounts, expressed as ‘If [context]… then [mechanism/outcome]’ statements, to be summarised in a table. These explanatory accounts will be used to refine the IPT and logic model and guide any needed adjustments to the intervention delivery platform.

For resource use data, we will calculate the potential cost of the intervention including training and staff time. These will be costed using information from the Personal Social Services Resource Unit.^44^ We will evaluate how the cost of the intervention might interact with different prison sizes and categories, and with different age profiles, and hence prevalence of dementia. The feasibility of conducting a budget impact analysis and suitable variables to go into a social return on investment analysis as part of a full evaluation study will be assessed. We will explore the suitability of using the EuroQol 5-Dimension, 5-Level version (EQ-5D-5L), DEMQoL or ASCOT to calculate the incremental cost per quality-adjusted life-year as part of a full economic evaluation alongside a future larger study. We will also look at any ceiling or floor effects for the summary utility score on these measures and report the proportion of participants who report each level in each domain.

### Patient and public involvement

We have developed a patient and public involvement and engagement (PPIE) group consisting of individuals who have previously lived in prison. PPIE co-applicants have been involved at all stages of the development of this research, for example attending meetings and drafting or reviewing written work, including the project proposal. PPIE collaborators co-produced the Plain English Summary to ensure it was clear, accurate and used appropriate language.

A group of individuals who experience dementia/MCI and are currently living in prison was also consulted. They were very supportive of the study and conveyed their struggles to receive appropriate assessment and care for their memory difficulties. None of them had received any social care input and stressed that prison is an acutely challenging environment in which to live with dementia. Not only did this inform the proposal design, but from these discussions, it also became apparent that these individuals would struggle to comprehend some of the research questions. This directly influenced our decision to include proxy versions of all outcome measures. It was also deemed important to let individuals select who should complete the proxy measure on their behalf, where possible. The plans for involvement and dissemination were developed in consultation with these two groups (one based in prison, and one based in the community).

The two PPIE groups will meet at least every 3 months. Two PPIE collaborators will also sit on the study’s advisory group. Participants will be reimbursed in line with NIHR guidance.

### Ethics and dissemination

The study will be conducted in full conformance with all relevant legal requirements and the principles of the Declaration of Helsinki, Good Clinical Practice (GCP), and the UK Policy Framework for Health and Social Care Research.^45^ Informed consent will be obtained as described in earlier sections.

This study received a favourable ethical opinion from the Wales REC 3 Research Ethics Committee in January 2025 (reference number 24/WA/0323). His Majesty’s Prison and Probation Service (HMPPS) National Research Committee approval was also granted in January 2025 (reference number 2024-1451).

Our planned outputs on completion of the study include stakeholder engagement events; at least two open-access papers; a series of short bespoke, visually attractive evidence briefings for commissioners, providers and practitioners; and newsletters for people living in prison. Presentations will be made at key academic and professional conferences.

## Supplementary material

10.1136/bmjopen-2025-115466 online supplemental file 1
