# Supplementary material for: Dementia and Mild Cognitive Impairment in Prison (DECISION) care pathway and training package: protocol for a realist-informed mixed-methods feasibility study
Source: BMJ Open. 2026 Feb 12;16(2):e115466. doi: 10.1136/bmjopen-2025-115466 (PMC12911847; doi:10.1136/bmjopen-2025-115466)
Supplement: online supplemental file 1 [file bmjopen-16-2-s001.docx]

Supplementary Table S1. CONSORT 2010 checklist of information to include when reporting a pilot or feasibility trial (adapted to reflect this is a study protocol for a non-randomised study)

| Section/Topic | Item No | Checklist item | Reported on page No |
| --- | --- | --- | --- |
| Title and abstract | | | |
|  | 1a | Identification as a pilot or feasibility study in the title | 1 |
|  | 1b | Structured summary of pilot study design, methods, results, and conclusions | 1-2 |
| Introduction | | | |
| Background and objectives | 2a | Scientific background and explanation of rationale for future definitive study | 4-5 |
|  | 2b | Specific objectives or research questions for pilot study | 5 |
| Methods | | | |
| Study design | 3a | Description of study design | 6 |
|  | 3b | Important changes to methods after pilot study commencement (such as eligibility criteria), with reasons | n/a (protocol) |
| Participants | 4a | Eligibility criteria for participants | 9, Table 1 |
|  | 4b | Settings and locations where the data will be collected | 6 |
|  | 4c | How participants will be identified and consented | 10-13 |
| Interventions | 5 | The intervention | 7-8 |
| Outcomes | 6a | Completely defined prespecified assessments or measurements to address each pilot study objective specified in 2b, including how and when they will be assessed | 13-16 |
|  | 6b | Any changes to pilot study assessments or measurements after the pilot trial commenced, with reasons | n/a (protocol) |
|  | 6c | If applicable, prespecified criteria used to judge whether, or how, to proceed with future definitive trial | n/a |
| Sample size | 7a | Rationale for numbers in the pilot study | 9-10 |
|  | 7b | When applicable, explanation of any interim analyses and stopping guidelines | n/a |
| Randomisation: |  |  |  |
| Sequence  generation | 8a | Method used to generate the random allocation sequence | n/a (non-randomised) |
|  | 8b | Type of randomisation(s); details of any restriction (such as blocking and block size) |  |
| Allocation  concealment  mechanism | 9 | Mechanism used to implement the random allocation sequence (such as sequentially numbered containers), describing any steps taken to conceal the sequence until interventions were assigned |  |
| Implementation | 10 | Who generated the random allocation sequence, who enrolled participants, and who assigned participants to interventions |  |
| Blinding | 11a | If done, who was blinded after assignment to interventions (for example, participants, care providers, those assessing outcomes) and how |  |
|  | 11b | If relevant, description of the similarity of interventions | n/a |
| Statistical methods | 12 | Methods used to address each pilot study objective whether qualitative or quantitative | 18-19 |
| Results | | | n/a (protocol) |
| Discussion | | | n/a (protocol) |
| Other information | | |  |
| Registration | 23 | Registration number for pilot trial and name of trial registry | n/a |
| Protocol | 24 | Where the pilot trial protocol can be accessed, if available | n/a (protocol) |
| Funding | 25 | Sources of funding and other support (such as supply of drugs), role of funders | 21 |
|  | 26 | Ethical approval or approval by research review committee, confirmed with reference number | 20-21 |
