# Supplementary material for: Dementia and Mild Cognitive Impairment in Prison (DECISION) care pathway and training package: protocol for a realist-informed mixed-methods feasibility study
Source: BMJ Open. 2026 Feb 12;16(2):e115466. doi: 10.1136/bmjopen-2025-115466 (PMC12911847; doi:10.1136/bmjopen-2025-115466)
Supplement: Supplementary file 2 [file bmjopen-2025-115466.reviewer_comments.pdf]

PEER REVIEW HISTORY

BMJ Open publishes all reviews undertaken for accepted manuscripts. Reviewers are asked to complete a checklist review form and are provided with free text boxes to elaborate on their assessment. These free text comments are reproduced below.

ARTICLE DETAILS

Title (Provisional)

Dementia and Mild Cognitive Impairment in Prison (DECISION) care pathway and training package: Protocol for a realist-informed mixed-methods feasibility study

Authors

Forsyth, Katrina; Buck, Deborah; Stalker, Kate; Allgar, Victoria; Shaw, Jennifer; Cowley-Sharp, Ryan; Hunter, Rachael; Lennox, Charlotte; O'Neill, Adam; Robinson, Catherine; Ware, Stuart; Robinson, Louise

---

This article was not externally reviewed at BMJ Open. Protocols that have been independently assessed prior to submission to BMJ Open are usually fast-tracked to publication on the grounds that further substantial changes will not be possible. This independent assessment will usually be external, independent review for both a substantial grant award from a non-commercial or government funder and ethics approval.
